# Risk of malignancy in 22q11.2 deletion syndrome

**DOI:** 10.1002/ccr3.880

**Published:** 2017-03-02

**Authors:** Toer Stevens, Jutte van der Werff ten Bosch, Marjan De Rademaeker, Ann Van Den Bogaert, Machiel van den Akker

**Affiliations:** ^1^ University of AntwerpAntwerpBelgium; ^2^ Department of Pediatric Hematology OncologyUZ BrusselBrusselsBelgium; ^3^ Centre for Medical Genetics, Reproduction and GeneticsReproduction Genetics and Regenerative MedicineUZ BrusselBrusselsBelgium; ^4^ Department of PediatricsQueen Paola Children's HospitalAntwerpBelgium

**Keywords:** Chromosome 22q11.2 Deletion Syndrome, Digeorge syndrome, malignancy, pineoblastoma, Velocardiofacial syndrome

## Abstract

22q11.2DS is a significant health problem because of its fairly high incidence. It is relevant to be vigilant regarding the diagnosis of cancer amongst 22q11.2 patients as there might be an increased risk, especially amongst patients with the 22q11.2 distal deletion syndrome.

## Introduction

The chromosome 22q11.2 deletion syndrome (22q11.2DS) occurs in approximately 1 of 3900 to 1 of 9700 children 1, 2. It is a multisystem disorder which is comprised of a broad variety of phenotypical syndromes that share a common genetic deletion. Amongst these syndromes are the DiGeorge syndrome, the Velocardiofacial syndrome, and the Cayler cardiofacial syndrome.

Major features can be summarized using the mnemonic CATCH‐22: cardiac abnormality (commonly interrupted aortic arch, truncus arteriosus, and tetralogy of fallot), abnormal facies, thymic hypoplasia (resulting in immune deficiency), cleft palate (velopharyngeal insufficiency), and hypocalcemia/hypoparathroidism 3. Additional findings may include learning difficulties, feeding disorders, early growth restriction, neurological conditions, structural abnormalities, hearing impairment, hypocalcemia, gut motility disorders, psychiatric, and hematological and autoimmune disorders 4, 5, 6, 7.

The 22q11.2 deletion results from variable‐sized heterozygous deletions of regions of 22q11, ranging from small deletions to deletions spanning approximately 3 megabases (Mb). The 22q11.2 deletion syndrome results most commonly from a 3 Mb deletion of the chromosome 22q11.2 region (about 90% of cases) and less common is a 1.5 Mb deletion (about 7%). In most cases, the deletions occur de novo but it may, however, also be inherited from either parent 6, 25. The prevalence of these de novo 22q11.2 deletions indicates an extremely high mutation rate within this region. Four distinct highly homologous blocks of low copy number repeats (LCRs), named LCR A‐D from proximal to distal, flank the deletion region which can lead to mispairing of the LCRs during meiosis resulting in unequal recombination events and hypothesized to cause the common recurrent rearrangement among patients with chromosome 22q11.2 deletion. With current treatment options, increasing numbers of patients with 22q11.2DS are reaching adulthood. To date, the true risk of malignancy in this patient group has not been defined.

## Case Report

**Figure 1:**
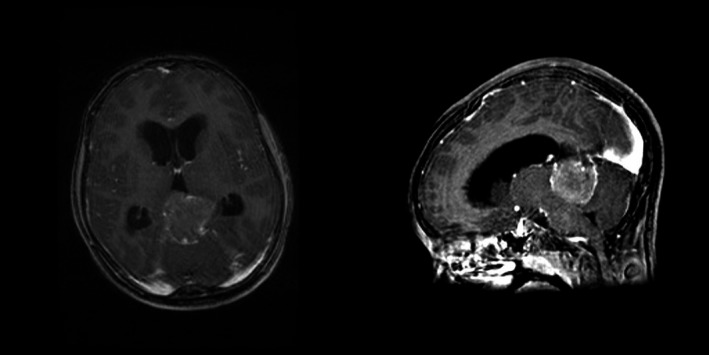
MRI of the brain: polynodular lesion in the epiphyseal region, partly calcified. Signs of hydrocephalus.

In May 2012, a 10‐year‐old male patient was diagnosed with a pineoblastoma (Fig. 1) with spinal metastases. He underwent a partial surgical resection and treated with adjuvant craniospinal radiation and chemotherapeutical therapy in accordance with the SJMB03 protocol. He received a total cumulative dose of 39.6 Gy (22 fractions) and an additional boost at his residual tumor which resulted in 59.4 Gy, final dose. Followed by three of the four cycles of high‐dose cyclophosphamide (4000 mg/m^2^/cycle), cisplatin (75 mg/m^2^/cycle), and vincristine (two 1.5 mg/m^2^ doses/cycle) and stem‐cell rescue. The fourth cycle was not given due to bone marrow failure.

Medical geneticists were consulted to evaluate his mild mental disability and facial dysmorphism. They ascertained that there had been a delay in speech development (no speech at 2.5 years of age), a moderate intellectual disability, a nasal speech, bad articulation and several foremost facial dysmorphism (small eye folds, mild upslanting of the palpebral fissures, small dysplastic auricles, attached ear lobes, short philtrum, and an upturned upper lip). Array CGH analysis was performed with a 44K Agilent array. A 2.5 Mb deletion was found at chromosome band 22q11.21 which is responsible for the physical and cognitive abnormalities. He was diagnosed with Velocardiofacial syndrome (DGS/VCFS). Genetic testing of the parents did not show this deletion. Like the majority of cases, the deletion occurred de novo. To date, 4 years after initial diagnosis, our patient is in good clinical health without disease recurrence.

## Discussion

Patients with 22q11.2DS have several features which increase the risk of malignancy. The thymic hypoplasia results in a range of T‐cell deficits, predisposing patients to more frequent or extensive infections with possible involvement of carcinogenic viruses such as Epstein–Barr virus or human papillomavirus, and possible also interfering with the orchestration and execution of the tumor surveillance. The most frequent cancer in primary immune deficiency patients is non‐Hodgkin lymphoma, but Hodgkin lymphoma and acute leukemia occur can also occur 8. The genetic defect itself can possibly play a role in the oncogenesis, by causing increased genomic instability, leading to double‐stranded breaks of the DNA causing genetic aberrations in genes involved in cell cycle and survival 9. Good examples are the RASopathies, diseases involving the RAS–MAPK signaling pathway, in which the overall malignancies risk is about ten‐fold higher than the general population 10.

Given its high frequency and the fact that with current treatment options, 90–95% of 22q11.2DS patients survive beyond young childhood 4, it is important to further define this risk. McDonald‐McGinn et al. 11 performed a multicenter cohort study which included 687 patients with the 22q11.2DS of which the majority was less than 10 years of age. Six cases of malignancy were identified which gave a relative risk of 0.9% as compared to an overall risk of malignancy in children under the age of 14 of 0.0034% (3.4/100,000/year). Even though one of the largest cohorts, these results are still confounded by population size given its overall low incidence. The authors postulated that the correlation between malignancy and 22q11.2DS is dependent on the severe T‐cell function impairment (associated with an increased risk of lymphoma), the state of chronic inflammation which could occur following the frequent infections predisposing to malignancy 12 and finally, the heterozygous deletion which generally includes the catechol‐O‐methyltransferase (*COMT*) gene. After deletion of this gene, its function as a detoxifier of certain environmental carcinogens is lost which relates to a mild increase in malignancy 13, 14, 15.

Up until now, 22q11.2DS is not considered to be associated with increased risk of malignancy. To evaluate the risk of malignancy in 22q11.2 deletion syndrome, a PubMed search was conducted in the English‐language literature using the keywords “22q11.2 Deletion Syndrome” or “DiGeorge Syndrome” or “Velocardiofacial Syndrome” and “cancer” or “malignancy.” No age limitation was used to include possible reports of patients with 22q11.2DS developing malignancy after childhood. The papers were reviewed, and the findings are summarized in Table 1.

**Table 1:** Overview of reported cases of malignancy amongst patients with 22q11.2DS

Reference	Malignancy	Demographic descriptives
Kim et al. (2015) 30	Lymphoproliferative mediastinal mass	F; 7 years
Pongpruttipan et al. (2012) 25	Pulmonal marginal zone lymphoma	F; 15 years
Finch et al. (2011) 22	Wilms tumor	M; 4 years
Itoh et al. (2011) 24	EBV‐associated T‐cell lymphoma	M; 25 years
Murray et al. (2011) 21	Temporal lobe pleomorphic xanthoastrocytoma	M; 16 years
McDonald‐Mcginn et al. (2006) 11	Neuroblastoma	M; congenital
	ALL	F; 8 years
	Hepatoblastoma	M; 3 months
	Hepatoblastoma	M; 15 months
	Wilms tumor	M; 4 years
	Thyroid carcinoma	F; 7 years
Ozbek et al. (2004) 20	Peripheral blood dysplasia	
Scattone et al. (2003) 19	Hepatoblastoma	M; 11 days
	Renal cell carcinoma	F; 6 years
Hong et al. (2001) 31	EBV+ DLBCL manifested with generalized lymphadenopathy	M; 7 months
Ramos et al. (1999) 23	B‐cell non‐Hodgkin's lymphoma involving mediastinal lymphnodes, brain, liver and kidneys	F; 10 months
Sato et al. (1998) 32	EBV+ DLBCL involving mediastinal lympnodes, mesenterium, lung, trachea, larynx, small intestine and liver	M; 7 months
Patrone et al. (1990) 18	Neuroblastoma	UK
Asamoto et al. (1977) 17	Glioma	UK
Tewfik et al. (1977) 16	Multiple squamous cell carcinoma	UK

F, female; M, male; UK, unknown; ALL, acute lymphoblastic leukemia; EBV, Epstein–Barr virus; DLBCL, diffuse large B‐cell lymphoma.

On the basis of the architecture of the 22q11.2 region, it was anticipated that other rearrangements, utilizing LCRs as recombination substrates and leading to different deletions might occur in the genomic region. Review of the literature revealed mostly de novo deletions of the LCR22‐4 to LCR22‐5 or LCR22‐4 to LCR22‐6 segment (distally to the critical region associated with DGS/VCFS) based on the existence of a breakpoint cluster region like (BCRL) module in each LCR, suggesting a potential substrate for NAHR‐mediated rearrangement in the distal 22q11.2. 26, 27. It is situated adjacent, but without the typically deleted regions in the conventional 22q11.2DS, leading to DGS/VCFS. Shay et al. 28 concluded that phenotypically there are similarities but also many characteristics distinct from 22q11.2DS. Several reports and reviews on the association between 22q11.2 distal deletion, amongst others containing the *SMARCB1* gene, and rhabdoid tumors are published. The malignancies related with 22q11.2 distal deletion, considering this being a different entity from 22q11.2DS, are intentionally not included in the table. Many microdeletion syndromes with identical deletions can share many common features, but can also show a high degree of variability among patients 29. It is therefore advisable that individuals with the characteristic “distal features,” but also with a less suggestive clinical 22q11.2DS phenotype, should also be tested for the 22q11.2 distal deletion. As nowadays array CGH is used to detect chromosomal anomalies and also to confirm a clinical suspicion of 22q11.2DS, the extension of the deletion can be delineated.

## Conclusion

More children with 22q11.2DS are reaching a higher age, and it is relevant to be vigilant regarding the diagnosis of cancer amongst 22q11.2 patients as there might be an increased risk. On top of that, there is a clear increase in the risk of malignancy amongst patients with the 22q11.2 distal deletion syndrome. Even though this is considered to be a separate entity, both clinical conditions have a broad variety of phenotypical presentations with many common features. It is therefore advisable to have a low threshold for testing for the 22q11.2 distal deletion.

## Authorship

TS, JvdWtB, MDR, and AVDB: were responsible for the writing of the manuscript. MvdA: was responsible for patient screening and supervised the writing of the manuscript.

## Conflict of Interest

The authors have no conflict of interests to disclose.
